# Antenatal corticosteroids is associated with better postnatal growth outcomes of very preterm infants: A national multicenter cohort study in China

**DOI:** 10.3389/fped.2022.1086920

**Published:** 2023-01-11

**Authors:** Tianhao Li, Wei Shen, Fan Wu, Jian Mao, Ling Liu, Yanmei Chang, Rong Zhang, Xiuzhen Ye, Yinping Qiu, Li Ma, Rui Cheng, Hui Wu, Dongmei Chen, Ling Chen, Ping Xu, Hua Mei, Sannan Wang, Falin Xu, Rong Ju, Zhi Zheng, Xinzhu Lin, Xiaomei Tong, The Chinese Multicenter EUGR Collaborative Group

**Affiliations:** ^1^ Department of Neonatology, Women and Children’s Hospital, School of Medicine, Xiamen University, Xiamen, China; ^2^ Xiamen Key Laboratory of Perinatal-Neonatal Infection, Xiamen University, Xiamen, China; ^3^ Department of Neonatology, The Third Affiliated Hospital of Guangzhou Medical University, Guangzhou, China; ^4^ Department of Pediatrics, Shengjing Hospital of China Medical University, Shenyang, China; ^5^ Department of Neonatology, Guiyang Maternal and Child Health Hospital·Guiyang Children’s Hospital, Guiyang, China; ^6^ Department of Pediatrics, Peking University Third Hospital, Beijing, China; ^7^ Department of Neonatology, Children’s Hospital of Fudan University, Shanghai, China; ^8^ Department of Neonatology, Guangdong Province Maternal and Children’s Hospital, Guangzhou, China; ^9^ Department of Neonatology, General Hospital of Ningxia Medical University, Yinchuan, China; ^10^ Department of Neonatology, Children’s Hospital of Hebei Province, Shijiazhuang, China; ^11^ Department of Neonatology, Children’ Hospital of Nanjing Medical University, Nanjing, China; ^12^ Department of Neonatology, The First Hospital of Jilin University, Changchun, China; ^13^ Department of Neonatology, Quanzhou Maternity and Children’s Hospital, Quanzhou, China; ^14^ Department of Pediatrics, Tongji Hospital, Tongji Medical College, Huazhong University of Science and Technology, Wuhan, China; ^15^ Department of Neonatology, Liaocheng People’s Hospital, Liaocheng, Shandong China; ^16^ Department of Neonatology, the Affiliated Hospital of Inner Mongolia Medical University, Hohhot, Inner Mongolia, China; ^17^ Department of Neonatology, Suzhou Municipal Hospital, Suzhou, China; ^18^ Department of Neonatology, The Third Affiliated Hospital of Zhengzhou University, Zhengzhou, China; ^19^ Department of Neonatology, Chengdu Women’ and Children's Central Hospital, School of Medicine, University of Electronic Science and Technology of China, Chengdu, China

**Keywords:** antenatal corticosteroids, enteral feeding, extrauterine growth restriction, nutrition, postnatal growth, very preterm infants, weight growth velocity, Z-score

## Abstract

**Introduction:**

Antenatal corticosteroids (ACS) administration is a standardized prenatal care for accelerating fetal maturation before anticipated preterm delivery, however, its effect on nutrition and growth is yet uncertain. This study aimed to examine if ACS application is associated with improvement in postnatal growth and nutrition in very preterm infants (VPIs).

**Methods:**

This was a secondary analysis of a multicenter prospective survey included infants born before 32 weeks gestation and admitted to 28 tertiary neonatal intensive care units throughout China from September 2019 to December 2020. Infants were divided into no ACS, partial ACS and complete ACS groups according to the steroids exposure. For infants exposed to antenatal corticosteroids, complete ACS was defined as receiving all doses of steroids 24 h-7 days before delivery, otherwise it was referred to partial ACS. The primary outcomes of postnatal growth were compared among the 3 groups. The multivariable regression analyses were applied to evaluate the association of different steroids coverage with postnatal growth and nutritional outcomes while adjusting for potential confounders. For each outcome, no ACS coverage was defined as the reference group. Data were presented as unstandardized coefficients or adjusted odds ratios with 95% confidence intervals, *P *< 0.05 (2-sided) indicated statistical significance.

**Results:**

Among 2,514 infants included, complete ACS, partial ACS and no ACS group accounted for 48.7% (1,224/2,514), 29.2% (735/2,514) and 22.1% (555/2,514), respectively. The median weight growth velocity was 14.6 g/kg/d, 14.1 g/kg/d and 13.5 g/kg/d in complete, partial and no ACS group respectively with significant difference (*P *< 0.001). In multivariable analyses, both complete and partial ACS coverage were associated with shorter cumulative fasting time, faster weight growth velocity, less dramatic decline in Z-score of weight, and lower incidence of extrauterine growth restriction [aOR (95%CI): 0.603 (0.460, 0.789) and 0.636 (0.476,0.851), respectively] when compared with no ACS. Moreover, the faster length growth velocity and earlier enteral feeding start time were observed only in infants with complete ACS coverage.

**Conclusions:**

Both complete and partial ACS are associated with better postnatal growth outcomes in very preterm infants. This efficacy appeared to be more obvious in infants exposed to complete ACS.

## 1. Introduction

Antenatal corticosteroids (ACS) administration, as a treatment to accelerate fetal maturation, has become a standardized practice to improve the survival and prognosis of premature infants (1–3). The beneficial effects of ACS on reducing neonatal mortality and major morbidities, such as neonatal respiratory distress syndrome (NRDS), intraventricular hemorrhage (IVH), and necrotizing enterocolitis (NEC) are well documented (1–3). It was reported ACS might also promote gastrointestinal tract maturation (4), but its potential effects on postnatal growth and nutrition of preterm infants have yet to be characterized.

Nearly 200,000 very preterm infants (VPIs), defined as gestational age (GA) less than 32 weeks, are born in China every year (5, 6), and the overall survival rate of VPIs is 87.6% (7). As steady improvements in outcomes have been reported, the quality of life for VPIs, especially their postnatal growth and nutritional status, has received increasing attention. A recent large-sample, multicenter prospective study in China (8) found that 47.3% of VPIs experience extrauterine growth restriction (EUGR), as evaluated by weight, and this incidence increased with decreasing GA, reaching 55.3% among VPIs born before GA 28 weeks, which is significantly higher than the reported EUGR incidence of 38% among VPIs in developed countries in 2012 (9). EUGR is associated with poor growth and neurodevelopment as well as with cardiometabolic alterations in childhood (10). Reducing the incidence of EUGR is a critical challenge for neonatologists in order to improve the short- and long-term prognoses of VPIs. A multi-center retrospective study shown that ACS appeared to have a small but significant protective effect against EUGR in preterm infants (11). Another retrospective cohort study also demonstrated the association between ACS and faster weight increase in very-low-birth-weight infant (12). According to the latest data from the Chinese Neonatal Network (CHNN), only 75.6% of VPIs received ACS exposure (7), which remained much lower than the 80%–90% reported in developed countries (13).

Given the lack of data concerning the association between ACS and nutrition of VPIs, we aimed to determine the hypothesis that ACS may improve postnatal growth and nutrition and reduce the incidence of EUGR among VPIs through a national, multi-center retrospective study.

## 2. Materials and methods

This was a secondary analysis of the data from a multicenter prospective survey (8) aiming to investigate the incidence and related factors of EUGR in VPIs during hospitalization across China conducted by the Chinese Multicenter EUGR Collaborative Group (trial registration: chictr.org.cn, number: ChiCTR1900023418). The clinical data were prospectively collected at 28 tertiary hospitals in 7 regions of China from September 2019 to December 2020. We retrospectively analyzed the data of the enrolled subjects for the present report.

### 2.1. Population

Infants born at GA <32 weeks, hospitalized for >14 days, and admitted to the participating neonatal intensive care units (NICUs) within 24 h after birth were eligible for this study. Every participating unit uniformly followed the clinical guidelines such as: European Consensus Guidelines on the Management of Respiratory Distress Syndrome - 2019 Update (1), Clinical practice of nutrition support in Chinese neonates (version 2013) (14), etc. and implemented clinical management accordingly. The exclusion criteria included: (1) major congenital malformation or genetic metabolic disease, (2) death during hospitalization, discharge against medical advice, (3) incomplete data, (4) rescue or repeat course of ACS.

Antenatal steroid regimens consist of betamethasone, two doses of 12 mg given intramuscularly 24 h apart, or dexamethasone, four doses of 6 mg given intramuscularly 12 h apart. Any ACS use was defined if at least one dose of steroids was administered. Complete ACS exposure was considered if all doses of steroids were received ≥24 h and <7days prior to delivery. Otherwise, it was defined as partial ACS. The guideline for ACS administration was uniform at all participating sites and consistent with the published recommendations (1, 2). Infants enrolled were divided into no ACS, partial ACS and complete ACS groups.

### 2.2. Outcomes

#### 2.2.1. Data collection and quality control

The following data were collected using a unified questionnaire: demographic characteristics of participants; maternal pregnancy complications (gestational hypertension and diabetes); postnatal growth and nutritional outcomes during hospitalization; and major morbidities and treatments during hospitalization. Demographic characteristics included GA at birth, birth weight, birth length, birth head circumference (HC), gender, mode of delivery, multiple birth, 1-minute and 5-minute Apgar score, small for gestational age (SGA) and ACS coverage.

The personnel in charge of data entry of each unit were uniformly trained. The EpiData database was established strictly according to the unified questionnaire, data of the case report form were recorded in double pairs, all participating units collected and uploaded the clinical data of preterm infants in time, and the database was locked after verification. The team leader maintained close contact with all participating units at any time point, checked the case records, and solved the possible problems in time.

#### 2.2.2. Assessed postnatal growth outcomes

During hospitalization, the body weight of VPIs was routinely measured by the attending nurses, using scales incorporated in incubators or external automatic scales. Beside body weight, anthropometric measures include length and HC, which were performed weekly until discharge. Length and HC were measured by an infantometer and a non-stretchable tape respectively. Postnatal growth indicators included body weight, length, and HC; greatest weight loss; days to regain birth weight and the incidence of EUGR.

The primary outcome of growth compared among the 3 groups included: growth velocity of weight, length and HC; change in Z-score of weight, length and HC from birth to discharge; and the incidence of EUGR. Weight growth velocity after regain of birth weight(g/kg/d) was calculated using an exponential model (15). Length and HC gain were calculated in centimeters per week from birth until discharge. Z-score were calculated from the updated Fenton growth charts (16). The change in Z-score (Zdischarge–Zbirth) was calculated to illustrate postnatal growth during hospitalization. EUGR was defined as weight below the 10th percentile at discharge.

#### 2.2.3. Assessed enteral and parenteral nutritional outcomes

The secondary outcome of nutritional status was evaluated among the 3 groups, including enteral feeding start time, days to full enteral feeding, cumulative fasting days, breastfeeding, accumulated energy intake for the first week, accumulative doses of amino acid and fat emulsions during the first week of hospitalization, and duration of parenteral nutrition. The time to reach full enteral feeding was the time required for oral feeding up to 150 ml/kg/d (17). Breastfeeding defined as start feeding with human milk by nasogastric tube or bottle.

#### 2.2.4. Assessed major morbidities and treatments during hospitalization

Major morbidities included: NRDS grade III–IV, moderate-to-severe BPD, hemodynamically significant patent ductus arteriosus (hsPDA), early- and late-onset sepsis, retinopathy of prematurity (ROP) requiring intervention, NEC (Bell stage ≥2) and IVH grade III–IV. The main treatments included: invasive mechanical ventilation (IMV) use; duration of IMV, non-invasive ventilation (NIV) and overall oxygen support; postpartum corticosteroid exposure; and duration of hospital stay. The diagnoses of these major morbidities were established by referring to Practical Neonatology (5th edition) (18).

### 2.3. Statistical analyses

All data were analyzed using SPSS 23.0 for Windows (SPSS Inc., Chicago, IL, United States). Categorical variables are shown as number of cases and percentages, and chi-square test was used for between-group comparisons unless the cell frequency was <5, in which case the Fisher's exact test was used. Distribution of continuous variables was assessed using the Shapiro–Wilk test. Non-normally distributed continuous variables are shown as median and interquartile range [M (IQR)], and the Mann–Whitney *U* test or Kruskal-Wallis H test were used for between-group comparisons. Pairwise comparisons between multiple groups were performed by Bonferroni test. Univariate analyses were performed for the comparisons of perinatal characteristics and nutritional outcomes among the cohorts.

Multivariable linear regression analyses were performed to assess the association of different ACS coverage with nutritional outcomes controlling potential confounders such as GA at birth, birth weight, gender, mode of delivery, multiple birth, 1-min and 5-min Apgar score, SGA, gestational hypertension, diabetes, breastfeeding, NRDS grade III-IV, moderate to severe BPD and IMV use. Linear relationship between continuous independent variables and dependent variables was verified by producing partial regression scatter plots. Durbin-Watson test was performed to assess the independence of residuals. The approximately normal distribution of standardized residuals was verified by histogram plots. The scatter plots composed by studentized residuals and unstandardized predicted values were produced to test the homoscedasticity of residuals. Tolerance and variance inflation factor were calculated to examine the multicollinearity of independent variables. Studentized deleted residuals, leverage values and cook's distances were calculated to investigate the significant outliers.

Multivariable logistic regression models were used to determine independent factors associated with EUGR adjusting for confounding factors as follows: GA at birth, birth weight, duration of hospital stay, gender, mode of delivery, multiple birth, 1-min and 5-min Apgar score, SGA, gestational hypertension, diabetes, breastfeeding, NRDS grade III-IV, moderate to severe BPD and IMV use. The Box-Tidwell method was used to check the linearity between the continuous independent variables and the logit conversion value of EUGR (yes or no). The Hosmer–Lemeshow test was conducted to determine the model's goodness of fit. Collinearity diagnostics was also conducted through tolerance and variance inflation factor calculation. Casewise diagnostics was used to identify the outliers outside the 2 standard deviations.

Considering ACS grouping are probably unequal spaced, we set dummy variable for ACS in all of the multivariable linear and logistic regression models, which made no-ACS group as reference, the other two groups were compared with it. Additionally, in subgroup analyses, the comparison between any ACS and no ACS was stratified by GA (24–27weeks, 28–30weeks and 30–31weeks, respectively). All statistical tests were two-tailed, and *P*-values <0.05 were considered statistically significant.

## 3. Results

**Figure 1:**
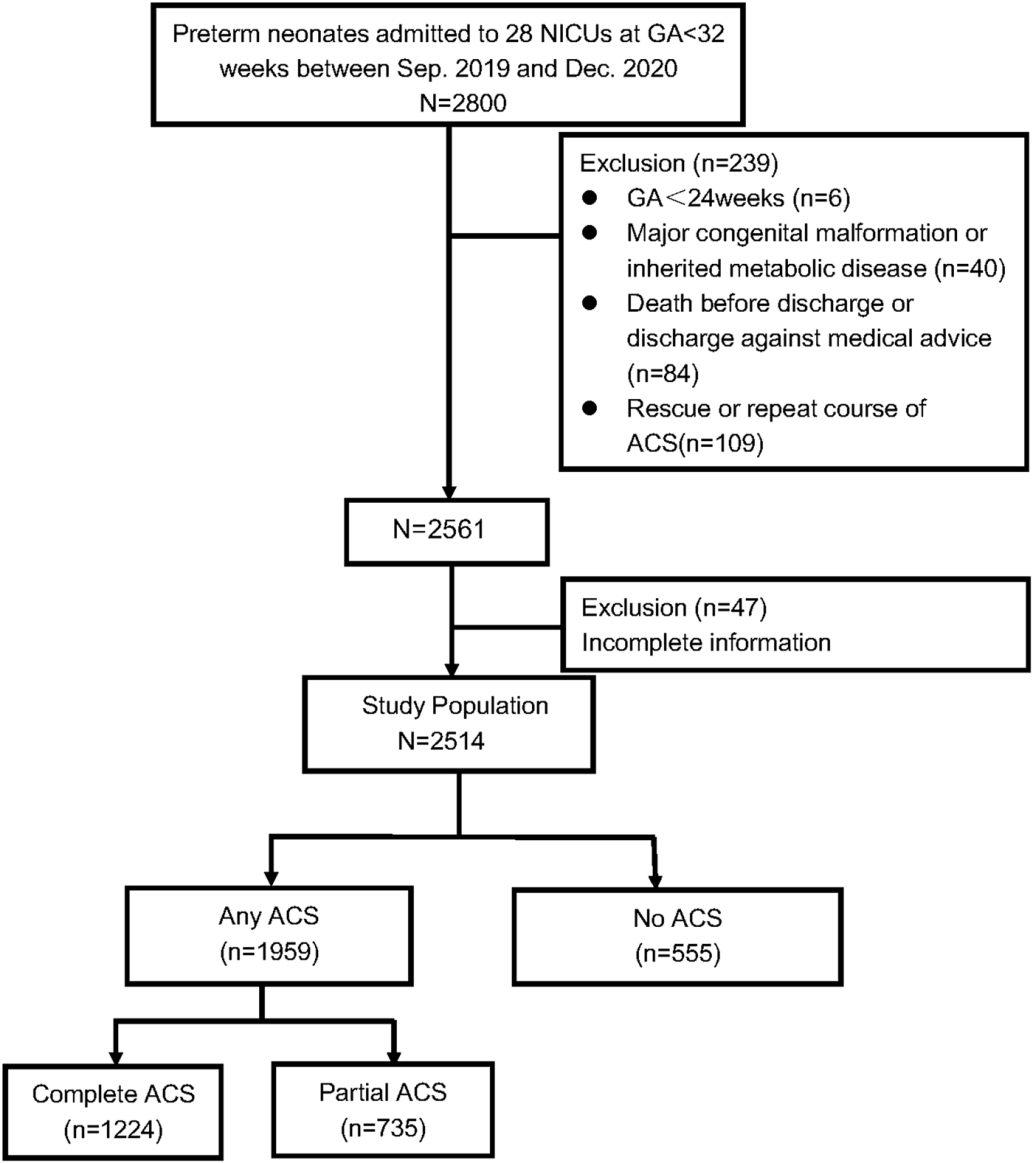
Flow chart of participants inclusion and exclusion. Abbreviations: ACS, antenatal corticosteriods; GA, gestational age; NICUs, neonatal intensive care units.

A total of 2,800 preterm infants with GA < 32 weeks were admitted during the study period. 6 infants aged <24 weeks at birth, 84 died during hospitalization or discharge against medical advice, 40 with severe congenital developmental abnormalities (such as gastrointestinal atresia, congenital megacolon, congenital diaphragmatic hernia, congenital pulmonary hypoplasia, microcephalus, fetal hydrops, etc.), inherited metabolic diseases or chromosomal abnormalities were excluded. A total of 47 infants were excluded because of missing data on weight, height, or HC. Finally, 2,514 VPIs were enrolled in the study. The flow chart of the included infants is shown in Figure 1.

### 3.1. Baseline characteristics according to ACS exposure

The study population included 2,514 VPIs with 77.9% (*n* = 1,959) infants received at least 1 dose of ACS. Of these population, complete ACS group accounted for 48.7% (*n* = 1,224) and partial ACS group 29.2% (*n* = 735). The incidences of cesarean section, multiple pregnancy, hypertension during pregnancy were significantly higher in the complete ACS and any ACS group than those in the no ACS group. Complete ACS was associated with lower median birth weight and lower male prevalence when compared to no ACS. There were no significant differences in GA, length, HC at birth; 1-min and 5-min Apgar score; SGA; and gestational diabetes among the 3 groups (Table 1). Complete ACS was associated with the lowest incidences of NRDS grade III–IV and moderate-to-severe BPD, while the likelihood of hsPDA, early- and late-onset sepsis, interventional ROP, NEC (Bell stage ≥2) and IVH grade III–IV were similar between groups. Infants exposed to Complete ACS had the lowest IMV use rate and shortest IMV duration, while infants without ACS had the highest IMV prevalence and longest IMV duration. Partial ACS exposed infants had the longest duration of non-invasive ventilation, but the duration of total oxygen therapy; postpartum corticosteroid exposure rate and length of hospital stay were similar between the groups (Table 2).

**Table 1:** Comparison of baseline characteristics.

	Any ACS (*n* = 1,959)	No ACS (*n* = 555)	*P_1_*	Partial ACS (*n* = 735)	Complete ACS (*n* = 1,224)	*P_2_*
Infant characteristics^a^						
GA at birth, weeks	30.1 (28.9,31.1)	30.1 (28.9,31.1)	0.942	30.1 (28.9,31.0)	30.1 (28.9,31.1)	0.742
GA at discharge, weeks	36.6 (35.4,37.9)	36.6 (35.6,37.9)	0.627	36.4 (35.4,37.9)	36.6 (35.6,37.9)	0.069
Birth weight, g	1,330 (1,130,1,530)	1,350 (1,130,1,560)	0.242	1,350 (1,150,1,550)	**1,300 (1**,**110,1**,520)*	0.019
Birth length, cm	39.0 (36.0,40.5)	38.1 (36.0,41.0)	0.947	39.0 (36.0,40.5)	39.0 (36.0,40.5)	0.390
Birth HC, cm	27.0 (26.0,28.5)	27.0 (26.0,29.0)	0.401	27.0 (26.0,28.5)	27.0 (26.0,28.5)	0.591
Male, n (%)	1,057(54.0)	320(57.7)	0.076	418(56.9)	**639(52.2)** *	0.036
1-min Apgar score	8 (7,9)	8 (7,9)	0.129	8 (7,9)	8 (7,9)	0.068
5-min Apgar score	9 (8,9)	9 (8,9)	0.495	9 (8,9)	9 (8,9)	0.247
SGA, n (%)	224(11.3)	66(11.8)	0.725	73(9.9)	151(12.3)	0.276
Maternal characteristics						
Cesarean delivery, n (%)	**1**,**247**(**63.7)**	304(54.8)	0.002	415(56.5)	**832(68.0)** *	<0.001
Multiple pregnancies, n (%)	**674**(**34.4)**	150(27.0)	0.006	246(33.5)*	**428(35.0)** *	0.007
Gestational hypertension, n (%)	**429**(**21.7)**	94(16.9)	0.014	134(18.2)	**295(24.1)** *	<0.001
Gestational diabetes, n (%)	342(17.3)	101(18.1)	0.637	121(16.5)	221(18.0)	0.632

ACS, antenatal corticosteroids; GA, gestational age; HC, head circumference; SGA, small for gestational age.

^a^ Reported as [M (Q1, Q3)], unless indicated otherwise.

*P*_1_ values were assessed between the any ACS group vs. no ACS group by the Mann–Whitney *U* test, *χ*^2^ test, or Fisher’s exact test.

*P*_2_ values were assessed among the complete, partial and no ACS groups by the Kruskal-Wallis H test, *χ*^2^ test, or Fisher’s exact test.

* Significantly different between no ACS group and ACS group.

**Table 2:** Comparison of major morbidities and treatments during hospitalization.

	Any ACS (*n* = 1,959)	No ACS (*n* = 555)	*P_1_*	Partial ACS (*n* = 735)	Complete ACS (*n* = 1,224)	*P_2_*
Major morbidities, n (%)						
NRDS grade III-IV	**274** (**14.0)**	110 (19.8)	0.001	**107 (14.6)** *	**167 (13.6)** *	0.003
Moderate-to-severe BPD	**297** (**15.2)**	109 (19.7)	0.011	116 (15.8)	**181 (14.8)** *	0.027
hsPDA	341 (17.4)	90 (16.2)	0.513	127 (17.3)	214 (17.5)	0.800
Early-onset sepsis	284 (14.5)	86 (15.5)	0.536	90(12.2)	194(15.8)	0.074
Late-onset sepsis	255 (13.0)	71 (12.8)	0.891	94(12.8)	161(13.2)	0.950
Interventional ROP^a^	62/1,955(3.2)	18/554(3.2)	0.927	24/733 (3.3)	38/1,222 (3.1)	0.219
NEC Bell stage ≥ 2	167 (8.5)	44 (7.9)	0.656	63 (8.6)	104 (8.5)	0.876
IVH grade III–IV^b^	40/1,923 (2.1)	9/549 (1.6)	0.468	19/728 (2.6)	21/1,195 (1.8)	0.176
Main treatments^c^						
IMV, n (%)	**910**(**46.5)**	322(58.0)	<0.001	371(50.5)*,**	**539(44.0)** *	<0.001
Duration of IMV	**0.0** (**0.0,3.0)**	1.0 (0.0,5.0)	<0.001	0.4 (0.0,3.0)*,**	**0.0 (0.0,3.0)** *	<0.001
Duration of non-invasive ventilation	15.0 (6.0,28.0)	14.0 (5.0,27.0)	0.056	16.0 (8.0,29.0)*,**	14.0 (6.0,28.0)	0.001
Duration of overall oxygen	30.0 (15.6,46.0)	29.0 (13.0,46.0)	0.450	30.0 (16.0,46.0)	29.0 (14.0,46.0)	0.084
Postpartum corticosteroid exposure, n (%)	268 (13.7)	82(14.8)	0.509	109 (14.8)	159 (13.0)	0.144
Duration of hospital stay	46.0 (35.0,60.0)	45.0 (35.0,60.0)	0.851	46.0 (35.0,57.0)	46.0 (35.0,60.0)	0.422

ACS, antenatal corticosteroids; NRDS, neonatal respiratory distress syndrome; BPD, bronchopulmonary dysplasia; hsPDA, hemodynamically significant patent ductus arteriosus; ROP, retinopathy of prematurity; NEC, necrotizing enterocolitis; IMV, invasive mechanical ventilation.

^a^ Calculated among infants who underwent fundus examination during hospitalization;.

^b^ Calculated among infants who underwent cranial ultrasound examination during hospitalization;.

^c^ Reported as [M (Q1, Q3)], unless indicated otherwise.

P1 values were assessed between the any ACS group vs. no ACS group by the Mann-Whitney *U* test, *χ*^2^ test, or Fisher’s exact test.

P2 values were assessed among the complete, partial and no ACS groups by the Kruskal-Wallis H test, *χ*^2^ test, or Fisher’s exact test.

* Significantly different between no ACS group and ACS group;.

** Significantly different between complete ACS group and partial ACS group.

### 3.2. Comparison of enteral and parenteral nutritional outcomes according to ACS exposure

Compared with no ACS, any ACS group demonstrated an earlier enteral feeding start time, a shorter cumulative fasting time and a higher breastfeeding rate. The accumulated energy intake for the first week was significantly higher in the any ACS group than in the no ACS group, but no significant differences were observed in the accumulative doses of amino acid and fat emulsions during the first week of hospitalization; the duration of parenteral nutrition support and the time to full enteral feeding (Table 3). Complete ACS coverage was associated with significantly earlier initiation of enteral feeding (21.0 h vs. 24.0 h, *P *< 0.001) and shorter median cumulative fasting days (1.45d vs. 2.1d, *P *< 0.001) than no ACS coverage. Partial ACS coverage was also associated with significantly reduced median cumulative fasting days (2.0d vs. 2.1d, *P *< 0.001) when compared with no ACS coverage (Table 3).

**Table 3:** Comparison of postnatal nutrition and growth outcomes during hospitalization^a^.

	Any ACS (*n* = 1,959)	No ACS (*n* = 555)	*P_1_*	Partial ACS (*n* = 735)	Complete ACS (*n* = 1,224)	*P_2_*
Nutritional outcomes						
Enteral feeding start time, h	**22.0** (**7.0,37.0)**	24.0 (11.0,48.0)	0.001	24.0 (11.0,44.0)	**21.0 (5.0,33.0)** *	<0.001
Cumulative fasting days, d	**1.8** (**0.8,4.0)**	2.1 (1.0,6.0)	<0.001	2.0 (1.0,4.5)*,**	**1.45 (0.6,4.0)** *	<0.001
Days to full enteral feeding, d	26.0 (18.0,36.0)	26.0 (18.0,35.0)	0.246	27.0 (18.0,37.0)	25.0 (17.0,35.0)	0.066
Breastfeeding, n (%)	**893**(**45.6)**	182(32.8)	<0.001	**342(46.5)** *	**550(44.9)** *	<0.001
Accumulated calories, kcal/kg^b^	**500.7** (**430.2,567.0)**	476.4 (400.0,553.4)	<0.001	**497.2 (425.0,572.2)** *	**504.9 (433.5,565.0)** *	<0.001
Accumulative amino acids, g/kg^b^	16.1 (13.3,18.5)	16.0 (13.5,18.0)	0.441	16.0 (13.3,18.2)	16.2 (13.3,18.8)	0.528
Accumulative fat emulsions, g/kg^b^	12.5 (10.0,15.0)	12.9 (10.0,15.0)	0.399	12.6 (10.2,15.0)	12.5 (9.6,15.0)	0.274
Duration of parenteral nutrition, d	21.0 (13.0,31.0)	21.0 (15.0,30.0)	0.554	21.0 (13.0,32.0)	21.0 (13.0,31.0)	0.728
Growth outcomes						
Greatest weight loss, %	**6.5** (**3.7,9.3)**	6.0 (3.2,8.8)	0.002	**7.0 (3.9,9.7)** *	6.3 (3.5,9.1)	<0.001
Days to regain birth weight, d	9.0 (7.0,12.0)	9.0 (7.0,12.0)	0.271	9.0 (7.0,12.0)	9.0 (7.0,11.0)	0.082
Weight at discharge, g	2,230 (2,050,2,490)	2,240 (2,020,2,500)	0.647	2,220 (2,030,2,470)	2,240 (2,050,2,500)	0.153
Length at discharge, cm	45.0 (43.3,46.3)	45.0 (43.0,46.0)	0.193	45.0 (43.0,46.0)	45.0 (43.5,46.5)	0.102
HC at discharge, cm	31.5 (30.7,32.5)	31.5 (31.0,32.8)	0.594	**31.5 (30.5,32.5)** **	32.0 (31.0,32.5)	0.004
Weight growth velocity, g/kg/d	**14.4** (**12.3,16.5)**	13.5 (11.6,15.7)	<0.001	14.1 (11.7,16.2)*,**	**14.6 (12.5,16.8)** *	<0.001
Length growth velocity, cm/w	**0.93** (**0.68,1.17)**	0.90 (0.63,1.14)	0.041	0.90 (0.66,1.15)	**0.94 (0.70,1.17)** *	0.011
HC growth velocity, cm/w	0.64 (0.48,0.78)	0.62 (0.47,0.78)	0.147	0.63 (0.47,0.76)	0.64 (0.48,0.79)	0.062
Change in Z-score of weight	**−1.05** (−**1.51,** −**0.62)**	−1.14 (−1.60, −0.74)	0.001	−**1.12 (**−**1.59,** −0.66)**	−**1.01 (**−**1.47,** −0.58)*	<0.001
Change in Z-score of length	−0.85 (−1.50, −0.18)	−0.90 (−1.68, −0.22)	0.087	−0.89 (−1.54, −0.21)	−0.82 (−1.46, −0.15)	0.089
Change in Z-score of HC	−0.78 (−1.50, −0.08)	−0.85 (−1.55, −0.10)	0.158	−0.84 (−1.52, −0.15)	−0.75 (−1.48, −0.04)	0.093

ACS, antenatal corticosteroids; HC, head circumference.

^a^ Reported as [M (Q1, Q3)], unless indicated otherwise.

^b^ During the first week.

*P*_1_ values were assessed between the any ACS group vs. no ACS group by the Mann-Whitney U test or *χ*^2^ test.

*P*_2_ values were assessed among the complete, partial and no ACS groups by the Kruskal-Wallis H test or *χ*^2^ test.

* Significantly different between no ACS group and ACS group.

** Significantly different between complete ACS group and partial ACS group.

Multivariable linear regression models revealed that significantly earlier initiation of enteral feeding[coefficient(95%CI): −5.808 (−10.818, −0.798)] can be seen only with complete ACS coverage. Reduced cumulative fasting time was observed both in infants exposed to complete and partial ACS (Table 4).

**Table 4:** Multivariable linear regression analyses for nutritional outcomes compared with No ACS group.

Nutritional outcomes	Unstandardized coefficients (B)	*P*	95%CI
Enteral feeding start time			
No ACS	0.0 (reference)		
Partial ACS	−1.729	0.533	(−7.167,3.709)
Complete ACS^a^	−5.808	0.023	(−10.818, −0.798)
Cumulative fasting days			
No ACS	0.0 (reference)		
Partial ACS^b^	−0.367	0.047	(−0.731, −0.018)
Complete ACS^a^	−0.777	<0.001	(−1.115, −0.438)
Weight growth velocity			
No ACS	0.0 (reference)		
Partial ACS^b^	0.500	0.009	(0.124,0.876)
Complete ACS^a^	0.640	<0.001	(0.293,0.986)
Length growth velocity			
No ACS	0.0 (reference)		
Partial ACS	0.021	0.320	(−0.021,0.064)
Complete ACS^a^	0.050	0.012	(0.011,0.089)
Change in Z-score of weight			
No ACS	0.0 (reference)		
Partial ACS^b^	0.077	0.026	(0.009,0.144)
Complete ACS^a^	0.170	<0.001	(0.108,0.232)

Data were adjusted for GA at birth, birth weight, gender, mode of delivery, multiple birth, 1-min and 5-min Apgar score, SGA, gestational hypertension, diabetes, breastfeeding, NRDS grade III-IV, moderate to severe BPD and IMV use.

ACS, antenatal corticosteroids; CI, confidence interval; NRDS, neonatal respiratory distress syndrome; BPD, bronchopulmonary dysplasia; IMV, invasive mechanical ventilation.

^a^ Complete ACS group showed significant outcomes when compared with no ACS group.

^b^ Partial ACS group showed significant outcomes when compared with no ACS group.

### 3.3. Comparison of postnatal growth outcomes according to ACS exposure

Compared with no ACS group, the greatest weight loss after birth was higher in the any ACS group, but the time to regain birth weight were similar between groups. The growth velocity of weight and length were significantly faster in the any ACS group, whereas the growth velocity of HC did not differ between groups. The ACS group showed a less dramatic decline in the Z-score of weight. At discharge, the body weight, length and HC of infants were similar between groups (Table 3). Complete ACS coverage was associated with the fastest weight growth velocity and least dramatic decline in the Z-score of weight among the 3 groups (*P *< 0.001 and *P *< 0.001, respectively). Partial ACS coverage also demonstrated a significantly promoted weight growth velocity compared to no ACS coverage. Growth velocity of HC and decrements in Z-score of length and HC did not differ among the 3 groups (Table 3).

Adjusting for potential confounders, multivariable linear regression analyses revealed that complete and partial ACS coverage were both associated with significantly faster weight growth velocity[coefficient(95%CI): 0.640(0.293,0.986) and 0.500(0.124,0.876), respectively] and less dramatic decrease in Z-score of weight[coefficient(95%CI): 0.170(0.108,0.232) and 0.077(0.009,0.144), respectively]. While improved length growth velocity[coefficient(95%CI): 0.050(0.011,0.089)] was observed only in infants exposed to complete ACS with statistical significance (Table 4).

### 3.4. Comparison of EUGR incidence according to ACS exposure

Infants exposed to any ACS were less likely to develop EUGR than those without ACS (46.1% vs. 51.5%, *P *= 0.022), especially for those born at GA 24–27weeks or with birth weight between 1,000 and 1,499 g (Table 5). Both complete and partial ACS exposure were associated with significantly lower incidence of EUGR compared to no ACS exposure. In subgroup analysis stratified by GA and birth weight, infants born at GA 24–27 weeks or with birth weight <1,000 g or between 1,000 and 1,499 g, who received complete ACS, demonstrated a significant reduction in EUGR compared to those without ACS. Partial ACS coverage also exhibited significantly lower likelihood of EUGR for infants born at GA 24–27 weeks than no ACS coverage (Table 5).

**Table 5:** Comparison of EUGR incidence according to ACS coverage.

Groups	Any ACS (*n* = 1,959)	No ACS (*n* = 555)	*P_1_*	Partial ACS (*n* = 735)	Complete ACS (*n* = 1,224)	*P_2_*
Overall, n (%)	**903 (46.1)**	286 (51.5)	0.022	**339 (46.1)** *	**564 (46.1)** *	0.022
GA at birth, weeks						
24 + 0 ∼ 27 + 6w, n/total (%)	**124/238 (52.1)**	44/66 (66.7)	0.035	**48/86 (55.8)** *	**76/152 (50.0)** *	0.038
28 + 0 ∼ 29 + 6w, n/total (%)	303/626 (48.4)	91/176 (51.7)	0.439	127/239 (53.1)	176/387 (45.5)	0.131
30 + 0 ∼ 31 + 6w, n/total (%)	476/1,095 (43.5)	151/313 (48.2)	0.134	164/410 (40.0)	312/685 (45.5)	0.066
Birth weight, g						
<1,000, n/total (%)	215/268(80.2)	52/59(88.1)	0.155	64/77(83.1)	**151/191(79.0)** *	0.046
≥1,000–<1,500, n/total (%)	**591/1**,**103(53.6)**	194/320(60.6)	0.026	235/423(55.6)	**356/680(52.4)** *	0.033
≥1,500, n/total (%)	97/588(16.5)	40/176(22.7)	0.059	40/235(17.0)	57/353(16.1)	0.162

ACS, antenatal corticosteroids; GA, gestational age; EUGR, extrauterine growth restriction.

*P_1_* values were assessed between the any ACS group vs. no ACS group by *χ*^2^ test.

*P_2_* values were assessed among the complete, partial and no ACS groups by *χ*^2^ test.

* Significantly different between no ACS group and ACS group.

### 3.5. Multivariable logistic regression models to determine independent factors associated with EUGR

Multivariable logistic regression models identified that both complete and partial ACS were associated with lower risk of EUGR [aOR(95%CI): 0.603(0.460,0.789) and 0.636(0.476,0.851), respectively] than no ACS (Table 6A). Subgroup analyses stratified by GA showed that a significant reduction in EUGR was observed in infants born at GA 24–27 weeks [aOR(95%CI): 0.480(0.244,0.942)] and GA 30–31 weeks [aOR(95%CI): 0.673(0.475,0.954)] with any ACS coverage compared to no ACS coverage, while the reduction in ACS exposed infants born at GA 28–29 weeks almost reached statistical significance (*P *= 0.05) (Table 6B).

**Table 6A:** Multivariable logistic regression models to determine independent factors associated with EUGR.

Variables	aOR	95%CI	*P*
ACS			
No ACS	1.0 (reference)		
Partial ACS	0.636	(0.476,0.851)	0.002
Complete ACS	0.603	(0.460,0.789)	<0.001
Male	1.545	(1.253,1.904)	<0.001
Breastfeeding	0.800	(0.650,0.984)	0.035
IMV	1.652	(1.299,2.102)	<0.001
Duration of hospital stay	1.060	(1.049,1.072)	0.001

Data were adjusted for GA at birth, birth weight, duration of hospital stay, gender, mode of delivery, multiple birth, 1-min and 5-min Apgar score, SGA, gestational hypertension, diabetes, breastfeeding, NRDS grade III-IV, moderate to severe BPD and IMV use.

No ACS group was set as referent group.

ACS, antenatal corticosteroids; CI, confidence interval; NRDS, neonatal respiratory distress syndrome; BPD, bronchopulmonary dysplasia; IMV, invasive mechanical ventilation; aOR, adjusted odds ratio.

**Table 6B:** Multivariable logistic regression models to identify the association between any ACS and EUGR with stratification by GA.

Variables	aOR	95%CI	*P*
Total			
No ACS	1.0 (reference)		
Any ACS	0.643	(0.503,0.822)	<0.001
GA 24 + 0–27 + 6w*			
No ACS	1.0 (reference)		
Any ACS	0.480	(0.244,0.942)	0.033
GA 28 + 0–29 + 6w			
No ACS	1.0 (reference)		
Any ACS	0.653	(0.427,0.999)	0.050
GA 30 + 0–31 + 6w*			
No ACS	1.0 (reference)		
Any ACS	0.673	(0.475,0.954)	0.026

Data were adjusted for GA at birth, birth weight, duration of hospital stay, gender, mode of delivery, multiple birth, 1-min and 5-min Apgar score, SGA, gestational hypertension, diabetes, breastfeeding, NRDS grade III-IV, moderate to severe BPD and IMV use.

No ACS group was set as referent group.

ACS, antenatal corticosteroids; CI, confidence interval; NRDS, neonatal respiratory distress syndrome; BPD, bronchopulmonary dysplasia; IMV, invasive mechanical ventilation; aOR, adjusted odds ratio.

* Subgroup analysis stratified by GA showed significant outcomes.

## 4. Discussion

Few studies have investigated the impacts of ACS on the postnatal nutrition of VPIs. In our study, both univariate and multivariable analysis showed that only complete ACS promoted earlier establishment of enteral feeding. Costalos et al. (4) studied the effect of ACS on gut peptides in preterm infants and found that gastrin levels in their ACS group were significantly higher both immediately after birth and after feeding, whereas motilin levels immediately after birth were similar between the two groups. After feeding, the motilin level in their ACS group was significantly higher than that in the non-ACS group, suggesting that ACS stimulates gastrin secretion in the fetus and may promote gastrointestinal development. Moreover, the remarkable increase in motilin after feeding may improve feeding tolerance and facilitate enteral nutrition. In our multivariable analysis, reduced cumulative fasting time was observed both in complete and partial ACS group, implying that ACS is associated with better enteral feeding tolerance.

In the present study, the ACS group demonstrated higher accumulated energy intake during the first week, but the accumulative doses of amino acids and fat emulsions during the first week of hospitalization did not differ significantly, suggesting that the increased calorie intake in the ACS group over the first week was mainly derived from early enteral nutrition. Our findings showed that ACS was associated with significantly lower incidence of moderate-to-severe BPD. The importance of enhanced enteral nutritional support in the prevention and management of BPD has been well documented (19, 20). Infants with BPD receive lower enteral energy intake than those without (21). Even if parenteral nutrition is used to compensate for the deficiency in enteral nutrition intake in the early postnatal period and to achieve the same total energy intake, the incidence of BPD is elevated among infants with insufficient enteral nutrition intake in the first 2 weeks after birth (22). A prospective cohort study showed that aggressive enteral nutritional support increases the rate of weight gain in VPIs with severe BPD (11.9 ± 2.9 vs. 8.9 ± 2.3 g/kg/d, *P *< 0.007) and reduces the incidence of EUGR (75.3% vs. 47.4%, *P *= 0.02) (23). Consequently, earlier establishment of enteral feeding and better feeding tolerance can reduce the incidence of BPD and EUGR. ACS promotes early aggressive enteral nutrition, specifically increasing oral calorie intake during the first week after birth, which plays an essential role in reducing the incidence of EUGR.

On the other hand, we found that ACS exposed VPIs were less likely to develop severe RDS and the reductions in the prevalence and duration of IMV may also contribute to the observed decrease in BPD in this population. Of note, our findings also showed that the most frequent need for NIV was observed in partial ACS group. Since RDS in partial ACS group was milder than no ACS group but severer than complete ACS group, the IMV duration in partial ACS group was shorter than no ACS group while more infants may have the chance to receive NIV support. Consequently, the prevalence of Moderate-to-severe BPD in partial ACS group was slightly lower than no ACS group, but the difference did not reach statistical significance in pairwise comparison.

Growth velocity during NICU hospitalization for preterm infants exerts a significant, and possibly independent, effect on neurodevelopmental and anthropometric outcomes (24). A retrospective cohort study (12) including 841 extremely low birth weight infants from 5 NICUs in Guangdong province from 2011 to 2014 found that single-course ACS was associated with higher rate of weight increase [adjusted coefficient(95%CI): 15.71(5.54,25.88)] compared to non-ACS, which was consistent with our results. In the present study, the greatest weight loss after birth among VPIs exposed to ACS was higher, which may be due to the fact that ACS increases the glomerular filtrate rate of preterm infants, thereby promoting early postnatal diuresis (25). However, the time to regain birth weight did not differ between groups, indicating a faster rate of birth weight recovery among infants with ACS. We also found that the weight growth velocity after regain of birth weight among infants exposed to ACS was greater, implying a “catch-up increase for weight” in the early postnatal period among infants with ACS may exist. In the *post hoc* analysis of various ACS coverage, infants with complete ACS exposure had the fastest weight growth velocity, while no ACS exposure was associated with the slowest weight growth velocity, suggesting that the beneficial effect of ACS on weight growth appeared to be dose-dependent. Despite the higher incidences of cesarean section, multiple pregnancy and hypertension during pregnancy in ACS group, our multivariable analysis with adjustment of these perinatal baseline characteristics identified that complete and partial ACS coverage both accelerated weight growth. Moreover, this effect appeared to be more evident in infants exposed to complete ACS.

The change in Z score of weight indicates the change in weight percentile for GA, reflecting the real situation of weight change. We always observe a dramatic decline in Z score of weight, length and HC from birth to discharge, which reflects a nutritional gap between intrauterine and extrauterine stages. Complete course of ACS appeared to alleviate the decline in Z-score of weight significantly and partial course was also observed to have a slight but significant protective effect, implying a truly beneficial effect of ACS on postnatal growth.

Monitoring of body length is an excellent means of assessing linear growth, which is also the most accurate indicator of lean body mass compared with weight or HC (26). We found that the fastest length growth velocity was seen only in infants with complete ACS coverage, implying that complete course of ACS is conducive to optimal linear growth among VPIs.

Our findings demonstrated that both complete and partial ACS coverage were independent protective factors against EUGR, which is consistent with a previous multi-center, retrospective analysis reported by Clark et al. (11) confirming ACS as a protective factor against EUGR [aOR(95%CI): 0.84(0.81,0.88)]. Of note, the lower male prevalence was observed in the complete ACS group, however, the evaluation of EUGR incidence was intrinsically divided by gender according to Fenton curves, which may not be influenced by the imbalanced distribution of gender. Moreover, to avoid the bias, we conducted the multivariable regression analyses adjusting the perinatal baseline characteristics including the covariant of male.

In subgroup analysis stratified by GA, the significant reduction in EUGR was observed in GA 24–27 weeks and GA 30–31 weeks, and even almost in GA 28–29 weeks. The previous literature (12) found that ACS promotes physical growth of neonates mainly in GA 28–31 weeks, while no significant difference was observed between groups born at GA < 28 weeks or ≥32 weeks. However, only 66 cases delivered at GA < 28 weeks were included in that study. The discrepancies in the subgroups stratified by GA observed in our study compared with previous research may be due to the low proportion of extremely preterm infants in the previous study.

Our findings showed that the ACS exposure rate among infants born at GA < 28 weeks was 78.3%, which reflects substantial progress over previously reported rates in China, including 39% in 2008–2012 (27), 35.8% in 2013–2014 (28), 54.5% in 2017 (29), and 71.7% in 2019 (7). However, compared with the data from the Eunice Kennedy Shriver National Institute of Child Health and Human Development Neonatal Research Network (NICHD) in 2018, the ACS administration rates in our study were approximately 14.3% lower for infants born at GA ≤ 25 weeks, 5.9% lower for those born at GA 26 weeks, and 16% lower for those born at GA 27 weeks (30). Therefore, standardized optimal ACS implementation in China for expected delivery of VPIs needs to be further popularized and promoted.

We addressed a significant and understudied topic about the association between ACS and postnatal growth of VPIs. Our results were derived from a large and recent multicenter cohort of VPIs who were cared for at 28 tertiary NICUs in 7 administrative regions throughout China, which makes our findings more generalizable. The data were prospectively collected and consistently defined with rigorous quality control. To our knowledge, this study provided one of the largest sample national-level assessment of the effectiveness of ACS on nutrition of VPIs in China, aiming to provide an evidence-based medical reference to optimize the nutrition for these patients.

The present study has several limitations. Firstly, this was a secondary analysis of prospectively collected data, although multivariable regression analyses were conducted, some potential confounders could not be eliminated. Secondly, this study excluded infants who were hospitalized for <2 weeks, those who died during hospitalization, potentially leading to bias in the investigation of the incidence of ACS use and its impact on clinical morbidities during hospitalization. Additionally, data on ACS coverage were recorded as ordinal categorical variable, the specific regimens such as timing, dose and dexamethasone or betamethasone were not available, which makes it impossible to comprehensively evaluate the efficacy of ACS. Future studies with larger population are needed to reassess the association between ACS and postnatal growth stratified by GA and birth weight. We hope to identify the specific population who will acquire the greatest growth benefit from ACS exposure. Whether certain subgroups of fetal growth restriction or multiple pregnancy would benefit likewise from ACS needs to be investigated. Furthermore, the long-term impacts of ACS on growth outcomes in childhood call for further research.

## 5. Conclusions

ACS promoted enteral nutrition in VPIs, accelerated their weight growth, alleviated their decline in Z-score of weight, and it was an independent protective factor against EUGR. This efficacy appeared to be more obvious in infants exposed to complete ACS. The ACS management in China still has substantial room for improvement. These implications indicate the importance of increasing the ACS administration rate and implementing standardized nutrition guidelines for VPIs in improving postnatal growth for this special population.

## Data Availability

The raw data supporting the conclusions of this article will be made available by the authors, without undue reservation.
